# Quantification of total fatty acids in microalgae: comparison of extraction and transesterification methods

**DOI:** 10.1007/s00216-014-8155-3

**Published:** 2014-09-16

**Authors:** Lillie R. Cavonius, Nils-Gunnar Carlsson, Ingrid Undeland

**Affiliations:** Food Science, Division of Life Science, Department of Chemical and Biological Engineering, Chalmers University of Technology, 41296 Gothenburg, Sweden

**Keywords:** Microalgae, Fatty acids, Bligh and Dyer extraction, Direct transesterification

## Abstract

Determination of microalgaes’ fatty acid content is often done with chloroform and methanol according to the Bligh and Dyer extraction, though faster methods exist. A number of comparisons between the Bligh and Dyer and faster methods have resulted in contradicting data, possibly due to differences in algae used and the different versions of the Bligh and Dyer method applied. Here, various forms of direct-transesterification (D-TE) and two-step transesterification (2-TE), including three versions developed in our lab, are compared with the original Bligh and Dyer (Can J Biochem Physiol 37: 911–917, 1959) extraction and two modifications thereof (Lee et al. J AOAC Int 79:487–492, 1996, and our own acidified version) on microalgae with different cell walls: *Isochrysis galbana*, *Nannochloropsis oculata*, and *Phaeodactylum tricornutum*. In total, fatty acid extracts from 11 methods were separated and quantified by gas chromatography with mass spectrometry. Results show that, for *N. oculata* and *P. tricornutum*, methods based on chloroform–methanol underestimated the fatty acid content compared with the 2-TE and D-TE methods, which gave similar results. Moreover, D-TE methods are faster than chloroform–methanol methods and use chemicals that are less toxic. Of the D-TE methods, the ones using hydrochloric acid and sulfuric acid recovered the most fatty acids, while boron trifluoride recovered slightly less. The main qualitative difference between the fatty acids recovered was that the chloroform–methanol methods recovered less saturated fatty acids in *P. tricornutum*.

**Electronic supplementary material:**

The online version of this article (doi:10.1007/s00216-014-8155-3) contains supplementary material, which is available to authorized users.

## Introduction

Microalgae are currently being investigated as possible sources of nutrition and biodiesel. In both applications, the main focus lies on the fatty acids produced by the microalgae. Therefore, methods for determining both the total quantity and type of the fatty acids in microalgae are needed. At present, there are many available methods, though there are only limited comparisons between them.

**Fig. 1:**
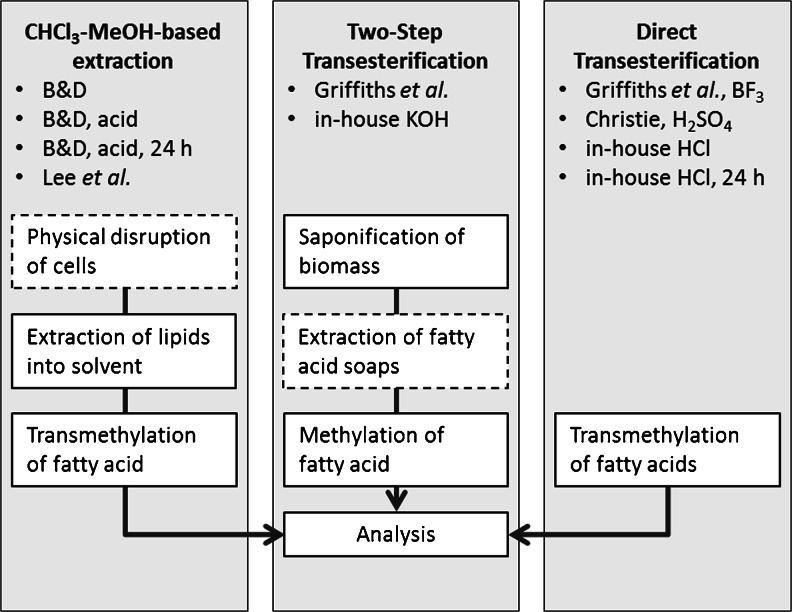
Simplified flowchart of the workflow in fatty acid analysis in microalgae by chloroform–methanol-based extraction, two-step transesterification, and direct transesterification. *B&D* Bligh and Dyer. The *dashed box* indicates an optional step, not necessarily present in all versions of the method

In 1959, Bligh and Dyer developed an extraction method based on chloroform and methanol, a solvent combination which proved to have a good ability to penetrate cells and recover total lipids from fish tissue [1]. The method is widely popular (currently cited over 31,000 times) and therefore allows for comparison of results from different studies, comprising many different matrices. However, it is important to stress that there are numerous modifications of the Bligh and Dyer method, some being presented as method modifications, others still being referred to as “Bligh and Dyer”. Although the Bligh and Dyer extraction is widely applied to microalgae, chloroform–methanol may not be the most appropriate solvent system for lipid extraction, due to differences in cell wall and lipid composition [2–18]. Furthermore, the Bligh and Dyer method was originally described as particularly good for tissue samples with low lipid content [1]. Later, Lee et al. demonstrated that increasing the chloroform-to-methanol ratio was a way to get accurate results for fish with >6 % lipid content [19]. Microalgae considered for biotech applications usually contain at least 10 % lipids. An overview of the Bligh and Dyer method’s main steps is shown in Fig. 1.

Two-step transesterification (2-TE) is a method in which biomass is treated with strong alkali, and fatty acids are subsequently methylated with an acidic catalyst. The alkaline condition breaks ester bonds, liberates the fatty acids [20], and possibly facilitates later extraction by degrading structures such as cell walls [21]. A method was developed in 1996 by which fatty acids were saponified and extracted from microalgae in a single step [22]. The resulting fatty acid extract can be further methylated and analyzed, as demonstrated by Burja et al. (2007) [23].

Direct-transesterification (D-TE) is a third method for analyzing fatty acids. Methods based on chloroform–methanol, as well as 2-TE methods require multiple steps before the fatty acids are methylated in the final step. D-TE applies the methylation agent directly to the biomass and thus reduces extraction steps. This technique was applied to microalgae already in 1990 [24]. Many different catalysts are currently used for D-TE, the most common being: hydrochloric acid (HCl), boron trifluoride (BF_3_), and sulfuric acid (H_2_SO_4_). However, according to the literature, there does not appear to be any agreement on which, if any, gives the most accurate results [20, 25–27].

To the best of our knowledge, it was not until 2007 that Burja et al. compared chloroform–methanol extraction with 2-TE and D-TE, specifically addressing microalgae [23]. The comparison was carried out on *Thraustrochytrium* sp., with the authors concluding that a miniaturized Bligh and Dyer gave the highest fatty acid yields [23]. Interestingly, Griffiths et al. compared various chloroform–methanol methods with a 2-TE on *Chlorella vulgaris, Scenedesmus* sp., and *Nannochloropsis* sp., concluding that the 2-TE method gave the highest yield and required less time and effort [28]. The conflicting results could be explained either by the different method versions used, or by differences in the algae’s cell walls. Therefore, further comparisons of fatty acid quantifying principles using morphologically different algal species is warranted.

Here, the aim was to compare three main groups of fatty acid-recovering methods by using three species of microalgae with different types of cell walls: *Phaeodactylum tricornutum*, with an organic cell wall [29, 30]; *Nannochloropsis oculata*, with its robust algaenan cell wall [31, 32]; and the comparatively fragile *Isochrysis galbana* [33]. Outcomes considered were total fatty acid yield, fatty acid profile, and the general practicality of the method. The three main principles for recovering fatty acids were: (1) chloroform–methanol-based extraction, (2) 2-TE and (3) D-TE. Within each main method group, several different versions were compared. For the Bligh and Dyer, this is justified by the many variations in circulation. 2-TE is known to give satisfactory results in previous studies and was included as a reference [23, 28]. We also present a new aggressive 2-TE method which was developed for disrupting and recovering fatty acids from algae with tough cells walls. Finally, different catalysts and versions of the D-TE were compared with find one that gives high yield, cuts down on toxic chemicals, and saves analysis time.

## Material and methods

### General preparation of microalgae

Microalgae were purchased dried from Necton (Olhão, Portugal, in 2012) and consisted of the following species: *I. galbana, N. oculata,* and *P. tricornutum*. It was confirmed that there was no water present (by freeze-drying). For all methods, 1 mg of the fatty acid C23:0 in chloroform was added as an internal standard to 12 mL glass tubes with Teflon screw-caps. Solvent was evaporated under nitrogen gas at 40 °C before 21 ± 4 mg of algal powder was weighed in. Since the areas of internal standard obtained in the chromatogram were in the same order of magnitude in all but one case, a second internal standard was not used. All fatty acid-recovering methods were carried out in triplicate.

### Chloroform–methanol extraction methods

#### Bligh and Dyer’s extraction

The original Bligh and Dyer method [1] was followed as closely as possible, although several modifications were required, since it was scaled down by a factor of 1,000. In brief, 20 mg algae were wetted for 60 min with 80 μL milli-Q water before 300 μL of chloroform–methanol (1:2) was added. Samples were vortexed for 2 min. Thereafter, 100 μL of chloroform was added; tubes were vortexed for 30 s; 100 μL milli-Q water was added to create a two-phase system and vortexed for 30 s more. Tubes were centrifuged (2,500×*g* for 6 min), the clear aqueous phase discarded, the chloroform phase recovered, and the residue re-extracted with 100 μL chloroform, centrifuging as above and pooling the recovered chloroform with the first portion. Chloroform extracts were methylated as described in “In-house methanolic-HCl transesterification” section and separated by gas chromatography-mass spectrometry (GC-MS) as described in “Analysis of fatty acids by GC-MS” section.

#### Bligh and Dyer’s acidic extraction

The Bligh and Dyer method was followed as described above (“Bligh and Dyer’s extraction” section), with our own modification where a two-phase system was created by adding 100 μL of 0.1 M HCl instead of milli-Q water. This precaution was taken to ensure that the fatty acids were protonated, making it more likely for the fatty acids to be present in the organic phase. The pH of the initial biomass was measured by suspending 20 mg of each type of algae in 200 mL milli-Q water; after sedimentation, ca. 10 μL of supernatant was spotted onto indicator paper (Macherey-Nagel Tri-test, Germany). Likewise, the pH of the aqueous phase remaining at the end of the extraction was measured by spotting 10 μL onto indicator paper. The organic phase was methylated as described in “In-house methanolic-HCl transesterification” section and separated as described in “Analysis of fatty acids by GC-MS” section.

#### Bligh and Dyer’s acidic extraction with overnight incubation

The Bligh and Dyer acidic method was followed as described above (“Bligh and Dyer’s acidic extraction” section), with the exception that the initial extraction with chloroform and methanol was extended to 24 h by incubating samples in the dark at room temperature on an orbital shaker running at 300 rpm. The rational for increasing the extraction time was to check if more fatty acids could be recovered, compared with the regular extraction time. The organic phase was methylated as described in “In-house methanolic-HCl transesterification” section and separated as described in “Analysis of fatty acids by GC-MS” section.

#### Lee et al.’s extraction

This method is a further development of the one published by Lee et al. [19]: To the microalgae, 8.0 mL of chloroform–methanol (2:1) was added, containing 0.05 % (*w/v*) of the antioxidant butylated hydroxytoluene (BHT; Fluka, Sweden). Tubes were vortexed for 60 s before adding 3.0 mL 0.5 % (*w/v*) sodium chloride solution. Tubes were then vortexed 15 s and centrifuged (2,000×*g* for 6 min). The organic phase was transferred to a new tube, methylated as described in “In-house methanolic-HCl transesterification” section and separated as described in “Analysis of fatty acids by GC-MS” section.

### Two-step transesterification (2-TE) methods

#### Griffiths et al.’s transesterification

This method was first published by Giffiths et al. [28], with minor changes applied here. Since dried microalgae were used, a water scavenger was not added here. Methanolic sodium methoxide, 0.5 M (Sigma-Aldrich, Sweden), 1.0 mL, was added, and the tubes were incubated at 80 °C for 20 min at 200 rpm shaking (the original method calls for 300 rpm, faster than our equipment could run). After allowing tubes to cool to room temperature, 1.0 mL of BF_3_, 14 % in methanol (Sigma-Aldrich, Sweden), was added to the samples and the incubation repeated. After allowing tubes to cool to room temperature, 0.4 mL of milli-Q water and 0.4 mL of hexane were added; tubes were vortexed and then centrifuged (2,500×*g* for 6 min). The organic layer containing the fatty-acid methyl-esters (FAMEs) was transferred to a new tube and prepared for GC-MS as described in “Analysis of fatty acids by GC-MS” section.

#### In-house two-step transesterification with KOH

To the microalgae, 4.0 mL of ethanol–methanol (ratio 3:2 *v/v*) was added, containing 0.05 % (*w/v*) of the antioxidant BHT. Potassium hydroxide (KOH) pellets, 0.4 g, were added and the tubes shaken until the pellets had dissolved (roughly 20 min). Tubes were incubated in a heating block at 70 °C for 120 min, with manual shaking every 10 min. After cooling the tubes to room temperature, 5.0 mL of toluene were added, and the tubes were vortexed briefly. To induce a two-phase system and protonate the fatty acids, 2.0 mL of 6 M HCl was added. Tubes were centrifuged at 2,000×*g* for 6 min, and the organic phase was transferred to a fresh tube and methylated as described in “In-house methanolic-HCl transesterification” section and separated as described in “Analysis of fatty acids by GC-MS” section.

### Direct-transesterification methods (D-TE)

#### Griffiths methanolic-BF_3_ transesterification

This method is a shortened version of Griffiths et al.’s method (“Griffiths et al.’s transesterification” section), omitting the incubation with sodium methoxide: Dry samples were incubated with 1.0 mL of BF_3_ at 80 °C and FAMEs extracted into hexane. FAMEs were prepared for GC-MS as described in “Analysis of fatty acids by GC-MS” section.

#### Christie’s methanolic-H_2_SO_4_ transesterification

This method is a scaled-down version of that previously published by Christie [26]. To 20 mg algae, 0.5 mL toluene and 1.0 mL of 1 % (*v/v*) H_2_SO_4_ in methanol were added. Tubes were flushed with nitrogen gas under manual agitation for 10 s before capping. Capped tubes were incubated at 50 °C for 17 h. Thereafter, 5.0 mL of 5 % (*w/v*) aqueous NaCl were added; tubes were vortexed briefly, and 5.0 mL of hexane was added. After vortexing, phases were separated by centrifuging (2,500×*g*, 6 min). The hexane phase was recovered, and the residue was re-extracted with a fresh aliquot of hexane. The two hexane phases were pooled. A small amount of the pooled hexane phase was extracted with water and the water’s pH tested on indicator paper (Macherey-Nagel Tri-test, Germany). Since the pH of the water phase was 6–7, the following steps described in the original method were omitted: The washing step with aqueous bicarbonate and subsequent drying over sodium sulfate. GC-MS was performed as described in “Analysis of fatty acids by GC-MS” section.

#### Lewis et al.’s methanolic-HCl transesterification

This method was first published by Lewis et al. and used by Burja et al. in their comparison of methods [23, 34]. Briefly, microalgae were incubated at 90 °C for 120 min with methanol–HCl–chloroform (10:1:1). Next, 1.0 mL of milli-Q water was added and the FAMEs extracted by adding 2.0 mL hexane–chloroform (4:1), vortexing and recovering the top layer, repeating the extraction a total of three times. The pooled organic layer was prepared for GC-MS, as described in “Analysis of fatty acids by GC-MS” section.

#### In-house methanolic-HCl transesterification

This in-house method is loosely based on that of Lepage and Roy [35]. This method was used both to directly methylate fatty acids in algae and to methylate fatty acid extracts from the chloroform–methanol methods and the in-house 2-TE. For direct methylation, 1.0 mL of toluene was added to the microalgae. For the other samples, i.e. methylation of extracts, solvent was first evaporated under a stream of nitrogen at 40 °C, and residues were re-suspended in 1.0 mL of toluene. Once toluene had been added, the procedure was identical for all samples: The 1.0 mL of freshly prepared 10 % (*v/v*) acetyl chloride in methanol was added, and tubes were incubated for 120 min at 70 °C. Tubes were allowed to cool, and the reaction was terminated by adding 0.2 mL of milli-Q water. FAMEs were extracted by adding 5.0 mL of petroleum ether-diethyl ether (4:1), vortexing briefly, centrifuging (2,500×*g* for 6 min) and transferring the organic (upper) phase to a fresh tube. GC-MS was performed as described in “Analysis of fatty acids by GC-MS” section.

#### In-house methanolic-HCl transesterification with overnight incubation

This method was applied only for direct-methylation purposes. The method is identical to the above ("In-house methanolic-HCl transesterification"), with the exception that the incubation at 70 °C for 120 min was exchanged for incubation in the dark at room temperature and shaking at 300 rpm for 24 h.

### Analysis of fatty acids by GC-MS

Solvent from extracts was evaporated under a stream of nitrogen at 40 °C and FAMEs re-suspended in 2,2,4-trimethylpentane. The extract was injected into an Agilent 7890 A GC system equipped with a J&W DB-wax column (30 m × 0.250 mm × 0.25 μm) and interfaced with a Agilent 5975 C triple-axis mass spectrometric (MS) detector in electron impact mode. Injection volume was 1 μL with a 15:1 split at an inlet temperature of 275 °C. The carrier gas was helium, with a fixed flow of 1 mL/min throughout the temperature program, which was as following—100 °C for 0 min, ramp at 4 °C/min to 205 °C, thereafter ramp at 1 °C/min to 230 °C, hold 5 min. Three external standards of FAME mixtures were used for identification of the different peaks in algal samples: GLC 463 (Nu-Chek prep, Inc., Elysian, USA), PUFA-3 (Supelco, Bellefonte, USA), C22:5 n-6 (Larodan, Malmö, Sweden). Fatty acids were quantified against the internal standard, summed, and expressed as milligrams FA per gram dry algae biomass.

### Statistical analysis

To investigate statistical differences between the means of different individual methods, Kruskal–Wallis test was applied, followed by Kruskal–Wallis stepwise step-down. Kruskal–Wallis is a non-parametric test and was used because the small number of replicates (*n* = 3, in most cases) did not allow to check if measurements were normally distributed. However, we expect a normal distribution and therefore report means and standard deviation. The software used was SPSS 19 (IBM). Means were considered significantly different when *p* ≤ 0.05.

## Results

### Comparison of chloroform–methanol extractions, 2-TE, and D-TE methods

**Fig. 2:**
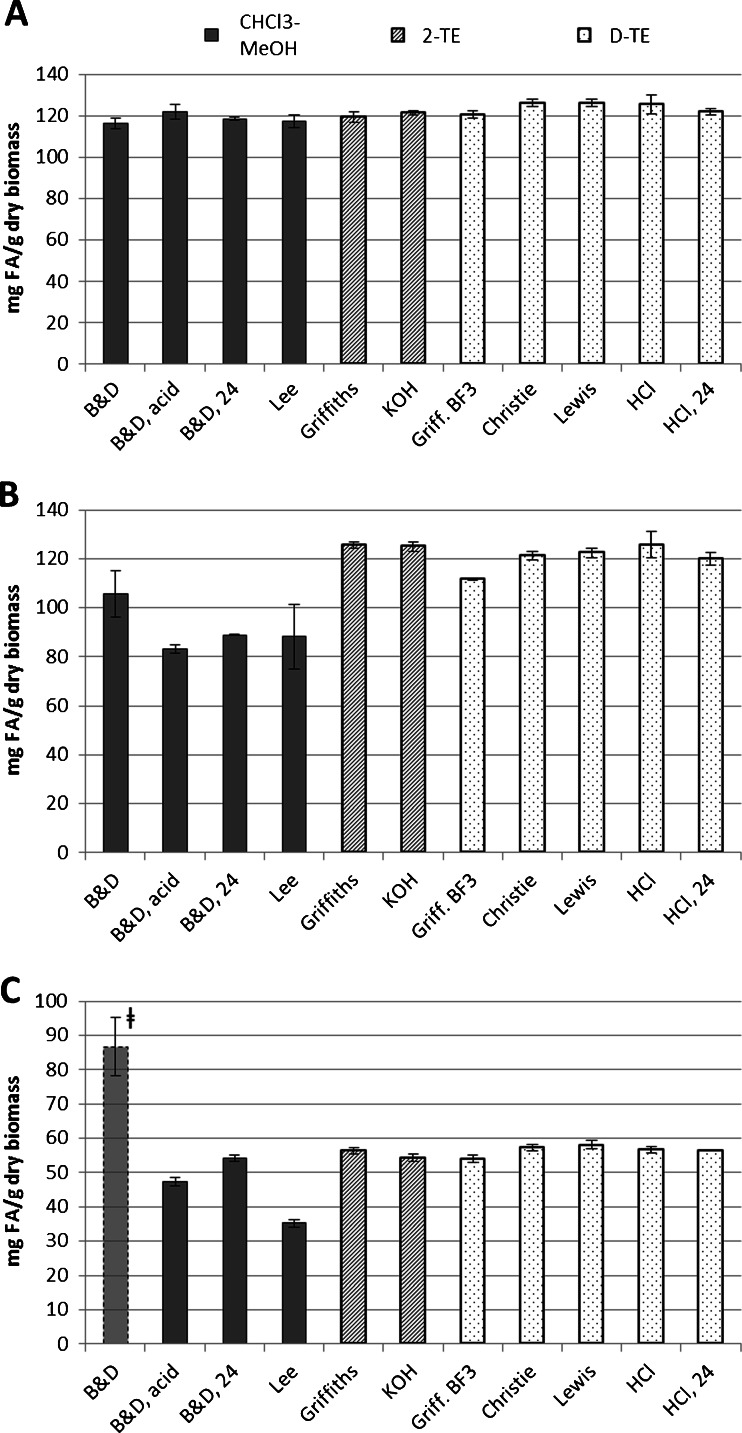
Total fatty acid content of three different algae, **A** *I. galbana*, **B** *N. oculata*, **C** *P. tricornutum*, as determinded by 11 different methods: *solid bars* represent chloroform–methanol extractions (“B&D” = Bligh and Dyer; acidified Bligh and Dyer; acidified Bligh and Dyer, overnight; Lee et al.), *cross-hatched bars* represent two-step transesterification (Griffiths et al.’s full method; in-house KOH method), and *speckled bars* represent direct-transesterification (short version of Griffiths et al.’ method with boron trifluoride; Christie’s method; Lewis et al.’s method; “HCl” = in-house acetyl chloride in methanol; in-house acetyl chloride in methanol, overnight). *Error bars* show ± standard deviation, *n* = 3 for all but HCl where *n* = 12. The high value for the Bligh and Dyer (*leftmost bar)* in **C** is an artifact caused by inadequate extraction of the internal standard

The chloroform–methanol-based extractions recovered significantly less total fatty acids than either the 2-TE or D-TE methods in *N. oculata* and *P. tricornutum*. The 2-TE methods and D-TE methods recovered approximately the same amounts of fatty acids from the same material. In *I. galbana*, the range of results was quite small (see Fig. 2a) with Lee et al.’s method recovering 117 mg FA/g dry algae and D-TE with either H_2_SO_4_ or HCl recovering 126 mg/g. Since all methods roughly agree, these results indicate that *I. galbana* is simple to analyze, and many methods therefore are valid. However, in *N. oculata*, there was a pronounced difference between chloroform–methanol extractions and the two groups of transesterification methods (see Fig. 2b), where the acidic Bligh and Dyer recovered as little as 83 mg/g, while Griffith et al.’s 2-TE and the in-house HCl D-TE recovered 126 mg/g. For some algae, obviously, the method of fatty acid extraction has a significant impact on the final results. In *P. tricornutum* (Fig. 2c), the chloroform–methanol-based extractions gave widely varying results. Although the Bligh and Dyer method appears to have recovered more fatty acids than any other method, this is an error caused by insufficient extraction of the internal standard (see “Acidification of Bligh and Dyer” section). Although increasing the extraction time of the acidified Bligh and Dyer to 24 h in *P. tricornutum* did lead to yields approaching that of the transesterification methods in this particular alga, the transesterification methods give a narrower range of results in all three tested microalgae. The differences between Bligh and Dyer’s and Lee et al.’s method on one side and the transesterification methods on the other was significant for both *N. oculata* and *P. tricornutum*.

For all three types of algae, the two 2-TE methods (Griffiths et al.’s method and the in-house KOH method) did not differ significantly in total FAMEs recovered. The 2-TE methods recovered approximately as much fatty acids as the D-TE methods. Although some significant differences were noted, no obvious trend emerged. For *I. galbana* (Fig. 2a), the results for the 2-TE methods were on the low end of the range for transesterification results while, for *N. oculata* (Fig. 2b), the 2-TE methods were on the high end of the transesterification results. For *P. tricornutum*, no strong differences between the 2-TE methods and D-TE methods emerged.

The D-TE methods recovered high amounts of FAMEs in all three microalgae. Amongst the D-TE methods, the BF_3_-method recovered significantly less fatty acids than any of the other D-TE methods. For all three species of microalgae, the methods of Christie, Lewis et al., and the in-house HCl method at 70 °C recovered among the most FAMEs. The in-house HCl-method at room temperature recovered significantly less total FAMEs than Christie’s method and Lewis et al.’s method in *I. galbana*.

### Acidification of Bligh and Dyer

After performing the Bligh and Dyer on *P. tricornutum*, it was suspected that the internal standard (C23:0) had not been fully extracted (see Fig. 2c), based on the standard’s small area in the chromatogram (data not shown), resulting in an unanticipated high recovery of FAMEs. Both for *N. oculata* and *P. tricornutum* (Fig. 2b and c), the total apparent FAMEs decreased when using diluted acid instead of milli-Q water to break the monophasic system. The pH of water suspensions of the various microalgae was as follows: *I. galbana* pH 6*, N. oculata* pH 7, and *P. tricornutum* pH 9; the pH of the aqueous phase remaining after the acidified extraction was pH 2, 3, and 5, respectively. Since the pH is expected to stay the same in the non-acidified Bligh and Dyer, the results indicate that the high pH in *P. tricornutum* and *N. oculata* samples caused the internal standard to be incompletely extracted, thereby overestimating the true FAME-content of the sample. It can be expected that other fatty acids present in the sample were also only partially extracted.

### Fatty acid profiles

**Fig. 3:**
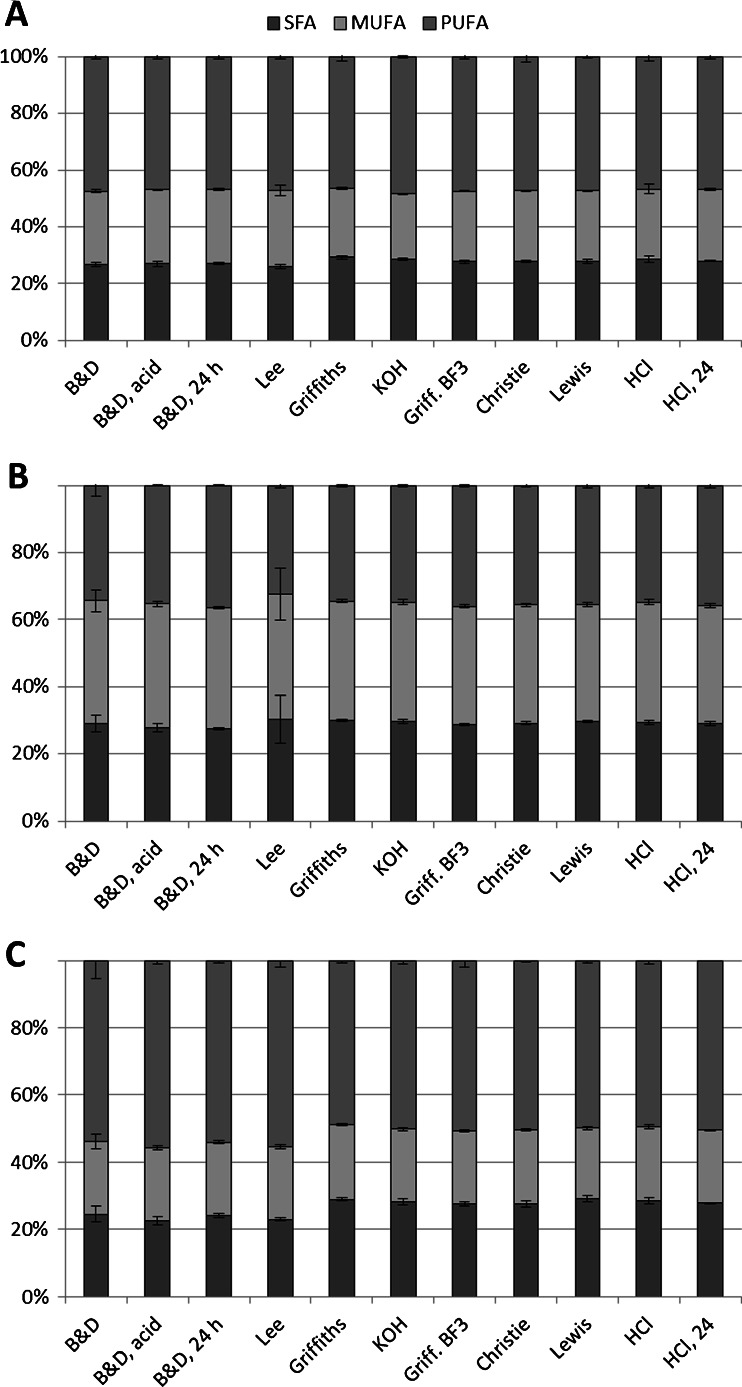
Saturated (*grey, bottom*), monounsaturated (*light grey, middle*), and polyunsaturated fatty acids (*medium grey, top*) as determined by different methods, in **A** *I. galbana*, **B** *N. oculata*, **C** *P. tricornutum*. Fatty acid groups are expressed as percent of total fatty acids. *Error bars* indicate ± standard deviation; *n* = 3 except for HCl, where *n* = 12. For method abbreviations, refer to Fig. 2

Fatty acid profiles (see Electronic Supplementary Material, Tables S1, S2, and S3) for the various methods were compared, to explore if methods showed preference for certain fatty acids. Though the percentage of many fatty acids was significantly different, no obvious pattern emerged. Instead of the entire patterns as a measure for preferential extraction of certain fatty acids, saturated fatty acids (SFAs), mono-unsaturated fatty acids (MUFAs), and polyunsaturated fatty acids (PUFAs) were summed and expressed as percentage of total extracted fatty acids (see Fig. 3). Although differences between different methods generally are quite small, the chloroform–methanol-based extractions recovered a significantly lower percentage of SFAs than the 2-TE and D-TE methods. In *I. galbana* and *N. oculata*, the decrease in SFA percentage in chloroform–methanol methods was offset by an increase in MUFA. In *P. tricornutum*, the decrease in SFA percentage is the most visible (Fig. 3c) and is offset by an increase in PUFA percentage.

## Discussion

Our results indicate that three D-TE methods (Christie’s method, Lewis et al.’s method, and the in-house HCl method) gave identical measures of total fatty acids on *I. galbana*, *N. oculata*, and *P. tricornutum*. Furthermore, two 2-TE methods (Griffiths et al.’s, in-house KOH) and the D-TE in-house overnight HCl-method gave results close to the three aforementioned methods. Methods based on chloroform–methanol-extraction recovered as much fatty acids as the 2-TE and D-TE methods for *I. galbana*. However, chloroform–methanol-extractions did not succeed in recovering as much fatty acids from *N. oculata* and *P. tricornutum*; this was expected, considering that (1) the algae had been chosen for their differences in cell wall composition and (2) other groups report *N. oculata* to be difficult to extract [36]. These results are in agreement with those of Griffiths et al., who compared various chloroform–methanol extractions to a 2-TE method on three microalgae including *Nannochloropsis* sp. and found the 2-TE method to consistently recover more fatty acids [28]. However, our results do not agree with Burja et al.’s study in which a Bligh and Dyer-based method recovered most oil from *Thraustochytrium* sp., closely followed by two different 2-TE methods, while Lewis et al.’s D-TE method recovered much less [23]. In our study, Lewis et al.’s method repeatedly gave high results, in contradiction to Burja et al.’s study. A possible explanation for the different results is that we quantified FAMEs from the Bligh and Dyer extraction by GC-MS instead of determining total lipids gravimetrically. While GC-MS allows quantification of specific molecules, gravimetric lipid determination may include varying amounts of hydrophobic and semi-hydrophobic impurities, depending on the solvent used to extract the lipids.

Lee et al.’s method, which was developed for fatty fish (>6 %) and uses a higher ratio of chloroform to methanol (2:1), did not recover more fatty acids than the original Bligh and Dyer method, even though the algae contained >5 % fatty acids. Lee et al. suggest themselves that the method is applicable to fish in which the fatty acids are mainly bound to triacylglycerols [19]. In the present study, the lipid classes were not assessed, i.e., the compounds to which the fatty acids were esterified were not determined. However, Ryckebosch et al. recently measured the lipid classes of *Isochrysis*, *Nannochloropsis*, and *Phaeodactylum*, showing that at least 40 % of the lipids extracted by their Bligh and Dyer method were phospholipids and glycolipids [37]. Our results also agree with Ryckebosch et al.’s earlier work where different solvent systems were compared on *Chlorella*, and the authors showed that a 1:1 ratio of chloroform–methanol recovered significantly more total lipids than a 2:1 ration of chloroform–methanol.

A further caveat of the chloroform–methanol extractions was apparent in *P. tricornutum*, where SFAs were recovered to a lesser degree than in the transesterification methods, indicating that the chloroform–methanol methods may be biased against SFAs. On closer inspection, the SFA C16:0 was recovered to a lesser extent. Other authors studying microalgae have found C16:0 to be present to the greatest extent in three lipids classes: (1) sulfoquinovosyldiacylglycerol [38, 39], (2) triacylglycerol [38], and (3) an unidentified phosopholipid [39]. Based on Ryckebosch et al.’s work [37], it seems likely that the chloroform–methanol extraction efficiently recovers the polar lipids—glycolipids and phospholipids—from *P. tricornutum* but recovers less of the more non-polar triacylglycerol-fraction. The recovery of all lipid classes by the various methods should be investigated in future work by adding, e.g., triacylglycerol, phospholipid, glycolipid, and fatty acid internal-standards to ensure that the methods are not biased towards any specific lipid class, such as fatty acid, used in this study.

In the Bligh and Dyer extraction, we noted the necessity to extract under acidic conditions in order to recover as much of the sample’s fatty acids as possible, which, in our case, was mainly the added internal standard. The presence of acid is expected to improve the extraction of free fatty acids for two reasons: (1) fatty acids become protonated, shifting their partitioning coefficient in favor of the organic phase and (2) the aqueous phase becomes more polar in the presence of ionic species (here: chloride and hydronium ions), resulting in non-polar molecules (such as lipids) being excluded to a greater degree, thus, in effect, shifting the partitioning coefficient of lipids in favor of the non-polar phase. It could be argued that acidic conditions should have been applied to Lee et al.’s extraction, but the area of the internal-standard in the chromatogram was similar to that of 2-TE and D-TE methods, indicating that the internal-standard had been recovered to the same extent; presumably, the addition of 0.5 % salt to the aqueous-phase was sufficient to make the aqueous-phase polar enough to recover the fatty acids.

The 2-TE methods gave high yields and small variations, yet it is difficult to justify the use of expensive chemicals such as sodium methoxide or long extraction times and many manipulations (e.g., requiring a full day’s lab work for 12 samples for the in-house KOH-method) when the D-TE methods give equally good results. At the onset of this study, we believed that more complete cell disruption would facilitate the extraction of cellular components; however, the in-house 2-TE method with KOH, specifically designed to achieve this, did not result in higher yield in all microalgae. Though the KOH method uses cheap reagents, it requires far more manipulation and time.

Among the D-TE methods, all except the BF_3_-method appeared to be equivalent in respect to the amount of total FAMEs recovered. Although the BF_3_-method recovered significantly less FAMEs in *N. oculata* and *P. tricornutum*, this is not necessarily caused by the catalyst, but rather by the short incubation time compared with other D-TE methods. It is possible that increasing the incubation time would improve the FAME-recovery for the BF_3_-method; however, we decided against proceeding with this catalyst, since it is hazardous to work with, expensive, and its shelf life is limited [20]. Since Christie’s D-TE with H_2_SO_4_, Lewis et al.’s method with HCl, and the in-house HCl-method all gave similar results, we would recommend any of these three for determination of FAMEs in microalgae. While the H_2_SO_4_-reagent is easy to prepare, the incubation takes longer than the other two.

The method developed by Lewis et al. uses methanolic-HCl with chloroform, a reagent which is easier to prepare than mixing methanol and acetyl chloride. The problem of chloroform sinking is elegantly circumvented by extracting the FAMEs into hexane. However, for those who wish to avoid chlorinated solvents, our in-house D-TE method comprising 10 % acetyl chloride in methanol presents an alternative. Acetyl chloride is less toxic than, e.g., boron trifluoride, though care must be taken when preparing the methylation reagent, since acetyl chloride reacts violently with methanol [26]. Although acetyl chloride is a chlorinated solvent in itself, the chlorine is ejected in reactions with water and alcohol, rendering less toxic products. Furthermore, we provide two versions of the in-house method which, here, gave similar results in the tested microalgae: Incubation can take place either at 70 °C for 120 min or at room temperature overnight. In both versions, it may be possible to shorten the incubation time, a possible focus for future work.

In conclusion, if the aim is to quantify total fatty acids from microalgae, the D-TE methods are faster and require less handling than do the 2-TE methods and the chloroform—methanol-based extractions, although results are generally similar. The D-TE methods utilizing hydrochloric acid or sulfuric acid catalysts recovered the most fatty acids of all methods here compared, with the added benefit that these D-TE methods do not necessarily require halogenated solvents. Lee et al.’s chloroform—methanol extraction recovered less fatty acids than any of the other methods in *N. oculata* and *P. tricornutum*, but, for *I. galbana*, all tested methods recovered roughly the same amount of fatty acids, indicating a significant matrix effect for different microalgae.

## Electronic supplementary material

Below is the link to the electronic supplementary material.

ESM 1 (PDF 84 kb)

## Abbreviations

2-TE: Two-step transesterification
BHT: Butylated hydroxytoluene [systematic name: 2,6-bis(1,1-dimethylethyl)-4-methylphenol]
D-TE: Direct transesterification
FAME: Fatty-acid methyl-ester
MUFA: Monounsaturated fatty acid
PUFA: Polyunsaturated fatty acid
SFA: Saturated fatty acid
