# Supplementary material for: Quantification of total fatty acids in microalgae: comparison of extraction and transesterification methods
Source: Anal Bioanal Chem. 2014 Sep 16;406(28):7313–22. doi: 10.1007/s00216-014-8155-3 (PMC4206773; doi:10.1007/s00216-014-8155-3)
Supplement: Supplementary file 1 — (PDF 84 kb) [file 216_2014_8155_MOESM1_ESM.pdf]

Analytical and Bioanalytical Chemistry

Electronic Supplementary Material

Quantification of total fatty acids in microalgae: comparison of extraction and transesterification methods

Lillie R Cavonius, Nils-Gunnar Carlsson, Ingrid Undeland

Table S1 Individual fatty acids expressed as mg/g dry weight in *Isochrysis galbana*; first row shows the mean, the second the standard deviation. Methods, in order of appearance: Bligh & Dyer; acidified Bligh & Dyer; acidified Bligh & Dyer, overnight; Lee et al.; Griffiths et al.’s full method; in-house KOH method; short version of Griffiths et al.’ method with boron trifluoride; Christie’s method; Lewis et al.’s method; “HCl” = in-house acetyl chloride in methanol; in-house acetyl chloride in methanol, overnight. SFA = saturated fatty acid, MUFA = monounsaturated fatty acid, PUFA = polyunsaturated fatty acid

**method**	**n**	**sum**	**C14:0**	**C16:0**	**C16:1 n7**	**C16:2 n4**	**C18:0**	**C18:1 n9**	**C18:1 n7**	**C18:2 n6**	**C18:3 n3**	**C18:4 n3**	**C20:1**	**C20:4 n6**	**C20:5 n3**	**C22:0**	**C22:5 n6**	**C22:6 n3**
			SFA	SFA	MUFA	PUFA	SFA	MUFA	MUFA	PUFA	PUFA	PUFA	MUFA	PUFA	PUFA	SFA	PUFA	PUFA
**Bligh & Dyer**	3	116	20	11	5,7	0,7	0,3	18	1,3	7,0	14	17	5,4	0,1	0,6	0,1	1,7	14
		2,5	0,8	0,2	0,13	0,02	0,01	0,3	0,03	0,1	0,2	0,3	0,1	0,03	0,02	0,03	0,02	0,4
**B&D, acid**	3	118	20	11	5,9	0,7	0,3	18	1,4	7,2	15	17	5,5	0,1	0,6	0,1	1,7	13
		1,2	1,0	0,1	0,05	0,00	0,00	0,3	0,02	0,1	0,2	0,2	0,1	0,02	0,02	0,02	0,1	0,2
**B&D, 24 hrs, acid**	3	118	20	11	5,8	0,7	0,3	18	1,4	7,1	15	17	5,6	0,1	0,6	0,1	1,7	13
		1,5	0,1	0,2	0,07	0,02	0,00	0,3	0,03	0,1	0,2	0,2	0,1	0,02	0,02	0,04	0,1	0,3
**Lee *et al.***	3	117	19	11	5,6	0,6	0,4	19	1,3	7,1	15	17	5,8	0,1	0,5	0,1	1,6	14
		3,0	0,5	0,8	0,09	0,02	0,2	1,8	0,05	0,1	0,2	0,2	0,2	0,1	0,02	0,01	0,1	0,2
**Griffiths *et al.***	3	121	22	13	6,1	0,7	0,3	18	1,4	7,5	14	17	3,9	0,1	0,6	0,1	1,8	14
		0,7	0,3	0,3	0,03	0,04	0,01	0,4	0,05	0,0	0,3	0,9	0,0	0,04	0,07	0,07	0,02	0,4
**KOH**	3	121	20	14	6,1	0,7	0,4	19	1,4	7,7	15	19	2,1	0,3	0,5	0,2	1,7	13
		1,1	0,3	0,1	0,08	0,02	0,04	0,2	0,02	0,2	0,2	0,2	0,05	0,03	0,02	0,07	0,1	0,03
**Griffiths, BF_3**	3	121	21	12	5,9	0,7	0,3	17	1,3	7,3	15	18	5,6	0,1	0,6	0,1	1,7	14
		1,9	0,6	0,2	0,06	0,01	0,01	0,2	0,02	0,04	0,1	0,3	0,1	0,02	0,01	0,01	0,04	0,4
**Christie**	3	128	22	13	6,4	0,7	0,3	18	1,4	7,6	15	19	5,7	0,1	0,6	0,1	1,7	16
		2,7	0,4	0,2	0,08	0,00	0,01	0,2	0,01	0,1	0,2	0,3	0,03	0,04	0,03	0,03	0,1	2,9
**Lewis *et al.***	3	126	22	13	6,1	0,7	0,3	18	1,3	7,7	15	19	5,9	0,1	0,6	0,1	1,8	15
		1,7	0,5	0,2	0,07	0,01	0,01	0,2	0,01	0,3	0,2	0,2	0,1	0,01	0,01	0,06	0,01	0,3
**HCl**	12	126	22	13	6,3	0,7	0,3	18	1,4	7,6	15	19	5,1	0,1	0,6	0,1	1,7	14
		4,7	1,1	0,5	0,2	0,01	0,01	0,6	0,04	0,2	0,5	0,8	1,6	0,04	0,1	0,1	0,1	0,5
**HCl, 24**	3	122	21	13	6,1	0,7	0,3	18	1,3	7,4	15	18	5,6	0,1	0,6	0,1	1,7	14
		1,4	0,2	0,1	0,07	0,01	0,01	0,2	0,02	0,1	0,2	0,2	0,1	0,1	0,05	0,1	0,03	0,4

Table S2 Individual fatty acids expressed as mg/g dry weight in *Nannochloropsis oculata*; first row shows the mean, the second the standard deviation. Methods, in order of appearance: Bligh & Dyer; acidified Bligh & Dyer; acidified Bligh & Dyer, overnight; Lee et al.; Griffiths et al.'s full method; in-house KOH method; short version of Griffiths et al.' method with boron trifluoride; Christie's method; Lewis et al.'s method; "HCl" = in-house acetyl chloride in methanol; in-house acetyl chloride in methanol, overnight. SFA = saturated fatty acid, MUFA = monounsaturated fatty acid, PUFA = polyunsaturated fatty acid

**method**	**n**	**sum**	**C14:0**	**C16:0**	**C16:1 n7**	**C18:0**	**C18:1 n9**	**C18:1 n7**	**C18:2 n6**	**C18:3 n3**	**C20:4 n6**	**C20:5 n3**
			SFA	SFA	MUFA	SFA	MUFA	MUFA	PUFA	PUFA	PUFA	PUFA
**Bligh & Dyer**	3	106	5,4	25	32	0,3	6,4	0,6	4,9	0,7	6,7	24
		9	0,5	2,1	2,7	0,0	0,6	0,0	0,4	0,07	0,7	2,4
**B&D, acid**	3	83	4,4	18	25	0,3	5,2	0,4	3,9	0,5	5,4	19
		2	0,5	0,4	0,7	0,0	0,0	0,0	0,0	0,02	0,0	0,1
**B&D, 24 hrs, acid**	3	89	4,6	20	26	0,3	5,5	0,5	4,3	0,5	6,1	22
		0	0,1	0,1	0,2	0,0	0,1	0,0	0,0	0,02	0,0	0,2
**Lee *et al*.**	3	88	3,9	22	24	0,9	8,7	0,6	3,5	0,5	5,1	19
		13	0,3	5,0	0,8	1,1	7,0	0,2	0,1	0,03	0,1	0,4
**Griffiths *et al*.**	3	126	6,1	31	37	0,4	7,3	0,6	5,4	0,7	8,7	28
		1,5	0,1	0,4	0,5	0,01	0,1	0,03	0,1	0,01	0,1	0,3
**KOH**	3	125	5,9	31	37	0,4	7,1	0,6	5,5	0,7	8,5	29
		2,0	0,2	0,6	0,7	0,01	0,1	0,01	0,2	0,01	0,05	0,2
**Griffiths, BF_3**	3	112	5,2	27	33	0,3	6,2	0,5	4,8	0,7	7,6	27
		0,4	0,1	0,3	0,5	0,01	0,1	0,01	0,04	0,02	0,1	0,2
**Christie**	3	121	5,7	29	35	0,4	7,0	0,3	5,3	0,7	8,4	29
		1,6	0,1	0,4	0,3	0,01	0,1	0,3	0,1	0,03	0,2	0,4
**Lewis *et al*.**	3	122	7,2	29	35	0,4	6,9	0,6	5,2	0,7	8,5	29
		1,8	0,1	0,3	0,7	0,01	0,1	0,01	0,1	0,04	0,1	0,7
**HCl**	12	126	6,4	30	37	0,4	7,1	0,6	5,4	0,7	8,7	29
		5,5	0,3	1,4	1,9	0,02	0,3	0,03	0,2	0,0	0,4	1,5
**HCl, 24**	3	120	5,8	29	35	0,4	6,9	0,6	5,3	0,7	8,5	28
		2,5	0,2	0,6	0,6	0,02	0,2	0,02	0,1	0,05	0,2	0,6

Table S3 Individual fatty acids expressed as mg/g dry weight in *Phaeodactylum tricornutum*; first row shows the mean, the second the standard deviation. SFA = saturated fatty acid, MUFA = monounsaturated fatty acid, PUFA = polyunsaturated fatty acid. Methods, in order of appearance: Bligh & Dyer *NB* concentrations falsely high, due to insufficient extraction of internal standard; acidified Bligh & Dyer; acidified Bligh & Dyer, overnight; Lee et al.; Griffiths et al.'s full method; in-house KOH method; short version of Griffiths et al.' method with boron trifluoride; Christie's method; Lewis et al.'s method; "HCl" = in-house acetyl chloride in methanol; in-house acetyl chloride in methanol, overnight. Where "N/A" appears, the samples were unsuccessfully spiked with C22:6 n3, making it impossible to reconstruct the original concentration; C22:6 n3 was not included in the sum in any of the methods

method	n	sum	C14:0	C16:0	C16:1 n7	C16:2 n4	C16:3 n4	C18:0	C18:1 n9	C18:1 n7	C18:2 n6	C20:4 n6	C20:4 n3	C20:5 n3	C22:6 n3	C24:0
			SFA	SFA	MUFA	PUFA	PUFA	SFA	MUFA	MUFA	PUFA	PUFA	PUFA	PUFA	PUFA	SFA
**Bligh & Dyer *NB***	3	87	6,2	13,9	16,5	3,1	9,3	0,4	0,8	1,4	1,6	1,1	0,5	31	N/A	0,8
		8,5	0,6	1,4	1,7	0,28	1,0	0,06	0,06	0,16	0,18	0,07	0,04	3,1		0,1
**B&D, acid**	3	47	3,1	7,5	9,3	1,8	5,4	0,2	0,4	0,8	0,9	0,6	0,2	17	1,0	0,1
		1,2	0,1	0,4	0,1	0,04	0,1	0,02	0,05	0,05	0,03	0,02	0,1	0,2	0,1	0,2
**B&D, 24 hrs, acid**	3	54	4,1	8,6	11	2,0	6,2	0,3	0,5	0,8	1,0	0,7	0,3	19	1,1	0,3
		1,0	0,1	0,2	0,2	0,04	0,1	0,01	0,02	0,02	0,03	0,01	0,01	0,3	0,02	0,1
**Lee *et al.***	3	35	2,5	5,3	7,0	1,4	4,4	0,2	0,3	0,5	0,6	0,3	0,2	13	N/A	0,2
		1,0	0,05	0,1	0,2	0,03	0,1	0,01	0,02	0,02	0,02	0,01	0,01	0,5		0,01
**Griffiths *et al.***	3	56	4,4	11	11	1,9	5,4	0,3	0,5	0,9	1,0	0,7	0,3	18	N/A	0,9
		0,9	0,1	0,1	0,2	0,02	0,1	0,02	0,03	0,02	0,02	0,03	0,01	0,2		0,03
**KOH**	3	55	3,8	10	10	1,9	5,8	0,3	0,6	0,9	1,0	0,7	0,3	18	N/A	1,0
		1,2	0,2	0,3	0,2	0,03	0,2	0,01	0,04	0,03	0,1	0,03	0,00	0,3		0,1
**Griffiths, BF_3**	3	54	4,0	10	10	1,9	5,6	0,3	0,6	0,8	0,9	0,6	0,3	18	N/A	1,0
		1,2	0,1	0,2	0,2	0,05	0,2	0,00	0,1	0,01	0,03	0,03	0,04	0,7		0,1
**Christie**	3	57	4,2	10	11	2,0	6,0	0,3	0,5	0,9	1,0	0,7	0,3	19	N/A	1,0
		1,0	0,3	0,1	0,2	0,04	0,1	0,02	0,02	0,01	0,04	0,03	0,01	0,2		0,1
**Lewis *et al.***	3	58	4,7	10	11	2,0	6,0	0,3	0,5	0,9	1,0	0,7	0,3	19	N/A	1,5
		1,2	0,3	0,2	0,3	0,05	0,1	0,01	0,02	0,01	0,02	0,02	0,01	0,3		0,03
**HCl**	12	57	4,3	10	11	2,0	5,9	0,3	0,5	1,0	1,0	0,7	0,3	18	1,0	1,2
		0,8	0,2	0,3	0,4	0,05	0,1	0,02	0,03	0,03	0,03	0,04	0,04	0,8	0,1	0,1
**HCl, 24**	3	56	4,1	10	11	2,0	5,9	0,3	0,5	1,0	1,0	0,7	0,2	19	N/A	1,2
		0,3	0,1	0,1	0,09	0,01	0,1	0,01	0,02	0,02	0,00	0,06	0,1	0,04		0,1
